# Color Doppler ultrasonography of an agitated solution is predictive of accurate catheter placement for a continuous popliteal sciatic nerve block

**DOI:** 10.1186/s13741-021-00229-w

**Published:** 2021-12-15

**Authors:** Clifford Bowens, Ignacio J. Badiola, Brian Frazer Scott Allen, Christopher Loredo Canlas, Rajnish Kumar Gupta, Lisa Michelle Jaeger, Eric Russell Briggs, John Matthew Corey, Yaping Shi, Jonathan Scott Schildcrout, Randall John Malchow

**Affiliations:** 1 Department of Anesthesiology, Vanderbilt University Medical Center, Nashville, TN USA; 2 Department of Anesthesiology and Critical Care, Perelman School of Medicine, University of Pennsylvania, Philadelphia, PA USA; 3 Department of Biostatistics, Vanderbilt University Medical Center, Nashville, TN USA

**Keywords:** Agitated solution, Color Doppler ultrasound, Perineural catheter placement, Popliteal sciatic nerve block, Ultrasound-guided regional anesthesia

## Abstract

**Background:**

Continuous peripheral nerve catheters (PNCs) have been shown to provide superior postoperative analgesia, decrease opioid consumption, and improve patient satisfaction compared with single injection techniques. In order to achieve success and reliability, accurate catheter positioning is an essential element of PNC placement. An agitated solution of normal saline, D5W, or a local anesthetic solution can be produced by the introduction of air to the injectate, creating air bubbles that can enhance ultrasonographic visualization and possibly improve block success.

**Methods:**

Eighty-three patients were enrolled. Ultrasound-guided continuous popliteal sciatic nerve blocks were performed by positioning the tip of a Tuohy needle between the tibial and common peroneal branches of the sciatic nerve and threading a catheter. An agitated local anesthetic solution was injected through the catheter, viewed with color Doppler ultrasound and video recorded. A peripheral block score (lower score = greater blockade, range 0-14) was calculated based upon the motor and sensory testing at 10, 20, and 30 min after block completion. The color Doppler agitation coverage pattern for the branches of the sciatic nerve was graded as follows: complete (> 50%), partial (> 0%, ≤ 50%), or none (0%).

**Results:**

The degree of nerve blockade at 30 min as judged by median (10th, 90th percentile) peripheral block score was significant for partial or complete color Doppler coverage of the sciatic nerve injectate compared to no coverage [3 (0, 7) vs 8 (4, 14); *p* < 0.01] and block onset was faster (*p* = 0.03). The block success was higher in groups with partial or complete coverage of the branches of the sciatic nerve vs no coverage (96% vs 70%; *p* = 0.02).

**Conclusions:**

Injection of an agitated solution through a popliteal sciatic perineural catheter is predictive of accurate catheter placement when partial or complete coverage of the sciatic nerve branches is visualized with color Doppler ultrasound.

**Trial registration:**

NCT01591603

## Background

Continuous peripheral nerve catheters (PNCs) have been shown to provide superior postoperative analgesia, decrease opioid consumption, and improve patient satisfaction compared with single injection techniques (Ding et al., 2015; Mariano et al., 2009; Ilfeld et al., 2003). Additionally, patients may be discharged home with portable infusion pumps that provide prolonged postoperative analgesia and reduce hospital length of stay (Klein et al., 2000; Salviz et al., 2013). Since these regional anesthesia infusion systems are the primary analgesic modality for these patients, it is paramount that they are reliable. In order to achieve success and reliability, accurate catheter positioning is an essential element of PNC placement.

When performing single-injection peripheral nerve blocks, local anesthetic spread around the target nerve is associated with successful blockade (Bowens Jr. et al., 2010; Perlas et al., 2009; Sala-Blanch et al., 2012). With PNC placement, the needle is similarly positioned but a catheter must be subsequently advanced through or off the needle into appropriate position. In the process of threading the catheter, the catheter may migrate away from the optimal perineural location. Furthermore, small-bore catheters are not well visualized with ultrasonography (Takatani et al., 2012; Ilfeld, 2011; Koscielniak-Nielsen et al., 2008; Swenson et al., 2008). To prevent catheter and block failure, providers need reliable methods to locate PNCs on ultrasound and confirm acceptable spread of injectate. Though methods have been developed to infer catheter tip location, no study to date has performed a comprehensive evaluation of any of these methods (Swenson et al., 2008; Dhir & Ganapathy, 2008; Kan et al., 2013; Elsharkawy et al., 2015). Dhir and Ganapathy introduced the concept of injecting an agitated solution and visualizing it with color Doppler ultrasound to confirm catheter position (Dhir & Ganapathy, 2008). An agitated solution of normal saline, D5W, or a local anesthetic solution is produced by the introduction air to the solution, creating air bubbles that can enhance ultrasonographic visualization. This is analogous to a “bubble study” used during echocardiography to detect a patent foramen ovale. We hypothesized that the coverage of the sciatic nerve branches with an agitated solution injection, visualized with 2D color Doppler ultrasound, would correlate with accurate placement of a continuous popliteal sciatic nerve block.

## Methods

The study was registered at ClinicalTrials.gov (NCT01591603), with the first author as the principal investigator. After obtaining IRB approval, 83 ASA physical statuses I to III adult patients scheduled for foot and ankle surgery were enrolled in a prospective study conducted at Vanderbilt University Medical Center and the Nashville Surgical Center. Written, informed consent was obtained from each participating patient. Patients with any of the following were excluded from the study: < 18 years old, body mass index (BMI) > 35, pregnancy, known lower extremity peripheral neuropathy, planned amputations, polytrauma patients, local anesthetic allergy, chronic pain (as defined by chronic pain syndrome diagnosis, implanted device for pain control, or use of long-acting opioids), and patients in whom communication was a problem. Patients, in-room anesthesia providers, surgeons, and recovery room nurses were blinded to the agitation coverage pattern. All data extraction was completed by the first author and all data was de-identified for statistical analysis.

The popliteal sciatic nerve blocks were performed in a pre-operative holding area primarily by senior level anesthesia residents and regional anesthesia fellows who were supervised by a regional anesthesiologist. A regional anesthesiologist was defined as a board-certified attending anesthesiologist on faculty at Vanderbilt University who spent the majority of their clinical time in the administration of regional anesthesia. The patient was placed in a supine position with routine monitors and provided supplemental oxygen. Midazolam and fentanyl or ketamine were administered and titrated to produce light sedation. After the operative leg was placed in a leg holder, the distal thigh and knee were sterilely prepped circumferentially and draped. Mask and sterile gloves were worn.

**Fig. 1:**
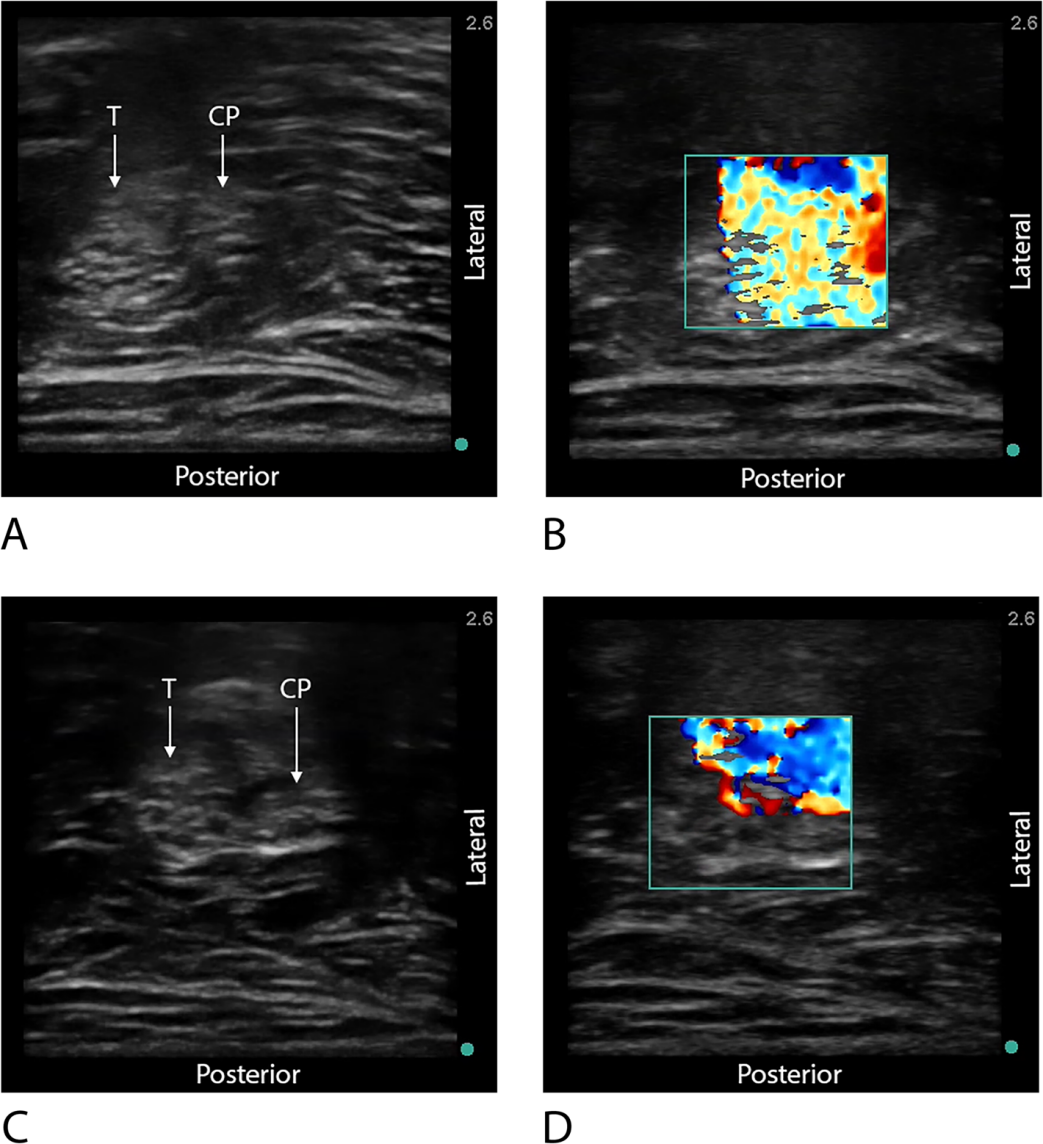
**A**, **C** After perineural catheter placement, ultrasound images displaying bifurcation of the sciatic nerve into tibial and common peroneal branches. Note, the catheter is not seen. **B** Doppler ultrasound image demonstrating complete sciatic nerve coverage, ≥ 50% of tibial and common peroneal branches are covered by the agitation solution. **D** Doppler ultrasound image demonstrating partial coverage of the sciatic nerve, < 50% of the tibial and common peroneal branches are covered by the agitation solution. T, tibial branch; CP, common peroneal branch

An ultrasound machine (S-Nerve, Fujifilm Sonosite, Bothell, WA) and ultrasound transducer (13-6 MHz L25x or 15-6 MHz HFL50x, Fujifilm Sonosite) were used in each case. After the ultrasound transducer was draped with a sterile sleeve cover, it was placed against the popliteal fossa of the knee. A transverse (short axis) view of both branches of sciatic nerve (tibial and common peroneal) was obtained that produced an image of the two branches at the bifurcation point (Fig. 1A, C). The skin and deep tissue was anesthetized with 1% lidocaine, 2-5 mL. An 18-gauge, 100 mm Contiplex Tuohy needle (B. Braun Medical Inc., Bethehem, PA) was advanced in-plane to a point between the tibial and common peroneal branches. Normal saline was injected through the Tuohy to confirm correct needle tip location. Adjustments were made as needed to reach the desired perineural position. A 20-gauge single-orifice, non-stimulating catheter (B. Braun Medical Inc., Bethehem, PA) was threaded either 1 cm or 5 cm (randomly determined) past the Tuohy tip. Once the final position was obtained, the catheter position was not adjusted. Using a 3-way stopcock, 1-3 mL aliquot(s) of an agitated solution consisting of 20 mL of 2% mepivacaine with 1:300 K epinephrine mixed with approximately 2 mL of air was injected through the catheter, visualized with 2D color Doppler ultrasound and video recorded for 6 s (Fig. 1B, D). The agitated solution was produced by rapidly mixing an air/local anesthetic solution back and forth between two syringes (20 mL and 5 mL) via a three-way stopcock, generating air bubbles within the solution (whitish-foam appearance). Before the bubbles had a chance to dissipate, the solution was injected (via 5-mL syringe) through the catheter. When color Doppler ultrasonography is used for visualization, the agitated solution produced a turbulent flow, readily visible within the Doppler window. After the initial 20 mL bolus was completed, an additional 10 mL of local anesthetic solution was injected through the catheter for a total volume bolus of 30 mL. Dermabond (Ethicon, LLC, San Lorenzo, Puerto Rico), a Lock-It Plus (Smiths Medical ASD, Inc., Keene, NH), and an occlusive dressing (Tegaderm; 3 M, St. Paul, Minnesota, USA) were used to secure the catheter.

A separate regional anesthesiologist, blinded to the details of catheter placement and Doppler appearance of the injectate, evaluated the nerve block. Sensory and motor function of the foot was assessed at 10, 20, and 30 min following completion of local anesthetic injection. Using ice, the following sensory nerve distributions were tested: sural (lateral foot), tibial (plantar foot), superficial peroneal (dorsal foot), and deep peroneal (first web space of foot). The following motor nerves were tested: tibial (plantar flexion), deep peroneal (dorsiflexion), and superficial peroneal (foot eversion). A 3-point scale was used to determine sensory or motor function: 2 = normal (normal sensation/full motor), 1 = partial function (hypesthesia/partial motor), and 0 = no function (no sensation/no motor). At the discretion of the operating room anesthesiologist, the block was used as the primary anesthetic or a general anesthetic was administered. Postoperatively, if the catheter was determined to be ineffective, it was adjusted or replaced for optimal patient care.

Data collected for each patient included gender; age; body mass index (BMI); American Society of Anesthesiologists (ASA) classification; diabetes mellitus; sedation medication; pain medication administered before, during, and after the surgical procedure; sciatic nerve visualization; local anesthetic injected; color Doppler video; Tuohy depth; catheter depth; sensory and motor groups blocked at 10, 20, and 30 min; surgical procedure; surgical region (hindfoot vs forefoot); length of surgery; and pain score (Numeric Rating Scale, range 0–10). Agitation coverage scoring was done for tibial branch, common peroneal branch, and the combination of the two branches. Adobe Premiere Pro CC 2018 (Adobe Systems, Inc., San Jose, CA) was utilized to capture the best image before and after agitation. Adobe Photoshop CC 2018 (Adobe Systems, Inc., San Jose, CA) image analysis tool was then used to calculate the percentage of area covered by the agitated solution for the tibial branch, common peroneal branch, and the combination of the two branches. The agitation coverage pattern was graded as follows: complete (> 50% coverage), partial (> 0%, ≤ 50%), or none (0% coverage).

The peripheral block score was computed by summing the sensory and motor values, which ranged from 0 to 14. A lower peripheral block score was associated with greater blockade. Block success was judged by postoperative recovery room pain score (≤ 3) or no need to treat pain with intra-operative or postoperative pain medications.

### Statistical analysis

Patient demographics and ultrasound characteristics were compared between agitation coverage groups using Kruskal-Wallis test and Pearson’s Chi-squared test for continuous and categorical variables, respectively. Continuous variables were summarized with the median (10th and 90th percentile) and categorical variables were summarized with percentages.

For the primary analysis, proportional odds models were used to examine the association between the peripheral block score at 30 min and the agitation coverage pattern for the tibial branch, common peroneal branch, and the combination of the branches. Since the agitation coverage pattern was not randomized and possibly influenced by confounding variables, an unadjusted and adjusted propensity score analysis was performed. The propensity model included patient gender, age, BMI, ASA classification, diabetes mellitus, and surgical region. In a secondary analysis, the onset of popliteal sciatic nerve blockade was compared between agitation groups by fitting proportional odds models. Robust sandwich standard error estimates were used to test whether there was a time-related decrease in peripheral block score (block onset difference) between agitation coverage groups. This analysis also was done with an unadjusted and adjusted propensity score.

All analyses were performed using R version 3.2.1, and two-sided significance levels of 5% were used to define statistical significance.

## Results

Eighty-one of 83 patients were included in the study. Two patients were excluded because there was no video record displaying agitation coverage. Ten of the 81 patients did not have a complete sensory and motor evaluation, which was due to insufficient time to complete the evaluation before the patient was transferred to the operating room. There were no significant differences among the groups with respect to gender, age, BMI, ASA classification, diabetes mellitus, surgical region, catheter depth, or sciatic nerve visualization (Table 1).

**Table 1:** Patient and ultrasound characteristics

Combined	None (***n*** = 10)	Partial (***n*** = 30)	Complete (***n*** = 41)	***P*** value
***Patient***				
Male	20.0	40.0	51.2	0.21
Age (years)	58 (51, 74)	52 (35, 61)	53 (29, 68)	0.20
Height (cm)	161 (156, 184)	170 (157, 180)	168 (155, 185)	0.32
Weight (kg)	83 (69, 88)	80 (63, 96)	80 (64, 104)	0.93
BMI	31 (26, 34)	28 (22, 34)	28 (23, 32)	0.31
ASA classification (I, II, III)	10.0, 60.0, 30.0	20.0, 56.7, 23.3	12.2, 65.9, 22.0	0.85
Diabetes mellitus (%)	0.00	3.3	9.8	0.43
Surgical region (hindfoot) (%)	60.0	66.7	78.0	0.47
Catheter depth (1 cm) (%)	60.0	63.3	75.6	0.47
***Ultrasound***				
Sciatic nerve visualization (good, fair, poor)	40.0, 30.0, 30.0	56.7, 36.7, 6.7	63.4, 26.8, 9.8	0.19

Median (10th, 90th) is used to summarize continuous variables and percentages are used for categorical variables, single value: **a**; multiple values: **a, b, c**. *P* values were calculated using the Kruskal-Wallis test for continuous variables and Pearson’s Chi-squared test for categorical variables. BMI, body mass index; ASA, American Society of Anesthesiologists

The median (10th, 90th percentile) peripheral block score at 30 min was significantly lower (greater blockade) in patients that had partial or complete sciatic nerve branch agitation coverage compared to no coverage [3 (0, 7) vs 8 (4, 14); *p* < 0.01] (Table 2) and block onset was faster (*p* = 0.03). The overall block success rate was significantly higher in groups that demonstrated sciatic nerve branch agitation coverage compared to no coverage (96% vs 70%; *p* = 0.02).

**Table 2:** Peripheral block scores (PBS) and block success

Comparison	PBS 10 (***N*** = 75)	PBS 20 (***N*** = 75)	PBS 30 (***N*** = 71)	Block success (***N*** = 81)
**Tibial and CP coverage**				
None (*n* = 10)	12 (8, 14)	9 (4, 13)	8 (4, 14)	7/10
Partial (*n* = 30)	8 (3, 13)	5 (0, 8)	1 (0, 6)	28/30
Complete (*n* = 41)	9 (5, 12)	6 (1, 9)	3 (0, 7)	40/41
*P* value	0.02^†^	0.01^†^	< 0.01^†^	0.02^††^
**Tibial and CP coverage**				
None (*n* = 10)	12 (8, 14)	9 (4, 13)	8 (4, 14)	7/10
Partial + complete (*n* = 71)	8 (4, 13)	5 (0, 9)	3 (0, 7)	68/71
*P* value	0.01^†^	< 0.01^†^	< 0.01^†^	0.02^††^

***N*** represents number of patients with peripheral block scores (PBS) at given time interval (lower score = greater blockade, range 0-14), block success includes all patients. ***n*** represents number of patients in each subgroup. Median (10th, 90th) is used to summarize continuous variables. Fractions, **a/b**, are used to summarize categorical variables, where **a** is the count and **b** is the subgroup size

^†^*P* values were calculated using the Kruskal-Wallis test for continuous variables

^††^*P* values were calculated using the Pearson’s Chi-squared test for categorical variables

All *P* values are significant. *CP* common peroneal

The propensity score adjusted odds ratio of obtaining greater blockade due to partial or complete tibial branch coverage compared to no coverage was 6.46 (95% CI, 1.77, 23.58; *p* < 0.01; Table 3). The adjusted odds ratio for partial or complete coverage for the common peroneal branch compared to no coverage was 1.92 (95% CI, 0.75, 4.89; *p* = 0.17). Compared to no coverage, partial or complete coverage of the combination of the tibial and common peroneal nerves was associated with a 14.53 (95% CI, 3.43, 61.58; *p* < 0.01) fold increase in the adjusted odds ratio of obtaining greater blockade.

**Table 3:** Odds ratio of greater blockade at 30 min

Comparison	Unadjusted		Adjusted	
	OR (95% CI)	***P*** value	OR (95% CI)	***P*** value
**Tibial branch coverage**				
None vs partial	9.70 (2.40, 39.22)	< 0.01*	6.32 (1.56, 25.65)	< 0.01
None vs complete	6.73 (1.82, 24.87)	< 0.01*	4.57 (1.21, 17.21)	0.02
None vs partial + complete	7.70 (2.17, 27.26)	< 0.01*	6.46 (1.77, 23.58)	< 0.01*
**Common peroneal branch coverage**				
None vs partial	1.63 (0.51, 5.20)	0.41	1.75 (0.54, 5.65)	0.35
None vs complete	1.82 (0.69, 4.75)	0.22	2.04 (0.74, 5.60)	0.17
None vs partial + complete	1.76 (0.71, 4.33)	0.22	1.92 (0.75, 4.89)	0.17
**Tibial and CP coverage**				
None vs partial	32.02 (6.73, 152.34)	< 0.01*	20.81 (4.38, 98.77)	< 0.01*
None vs complete	13.49 (3.22, 56.62)	< 0.01*	9.08 (2.09, 39.47)	< 0.01*
None vs partial + complete	17.71 (4.35, 72.22)	< 0.01*	14.53 (3.43, 61.58)	< 0.01*

Unadjusted and propensity score adjusted odds ratios (95% CI) and *P* values were calculated using proportional odds models. Propensity score adjustment was based on patient gender, age, BMI, ASA status, diabetes mellitus, and surgical region

*Denotes significance

*CP* common peroneal

On catheter day 1 (post-operative day 0), 6 nonfunctioning catheters were replaced, 3 from the no coverage group, 2 from the partial coverage group, and 1 from the complete coverage group. On catheter day 2, all catheters were working except 3 catheters that had become dislodged, 2 from the partial coverage group and 1 from the complete coverage group. There was no significance associated with the rate of dislodgement between the agitation groups.

## Discussion

As a method to suggest adequate PNC placement, an agitated solution with Doppler visualization is a promising adjunct to traditional and sometimes challenging confirmation methods such as catheter visualization and spread visualization without Doppler. In this study, we demonstrated that the degree of color Doppler ultrasound enhancement of sciatic nerve area covered by an agitated injectate was associated with faster onset and higher block success for a popliteal sciatic PNC. In addition, tibial branch coverage compared to common peroneal branch coverage showed a higher correlation with greater blockade. The tibial branch of the sciatic nerve is larger than the common peroneal branch; therefore, better coverage of the tibial branch may have led to easier subsequent spread of local anesthetic to the smaller common peroneal branch. Ninety-six percent (68/71) of the catheters that had partial or complete coverage were clinically successful. Seventy percent (7/10) of catheters that did not show agitation coverage were still clinically effective. Therefore, positive agitation coverage correlated well with successful catheter placement (high sensitivity), but zero agitation coverage did not necessarily correlate with catheter failure (low specificity). In this study, the catheter was not allowed to be moved after initial placement, but in routine clinical practice a perineural catheter could be adjusted to improve agitation coverage. There were no adverse clinical events associated with the injection of the agitated solution during the study.

The “Methods” section describes one strategy to create an agitated solution requiring only the addition of a 3-way stopcock to a PNC kit. An agitated solution also can be created, using the air/local anesthetic solution previously described, with a single 20- or 30-mL syringe by pulling the plunger back several times and releasing it in rapid succession to produce the agitated solution. Other methods of catheter localization that exist, visualizing either a small 20-gauge catheter or hypoechoic perineural volume expansion, can be challenging (Takatani et al., 2012; Johns et al., 2014). Agitated injection paired with color Doppler is an imminently practical solution to locating a PNC after placement, requiring readily available materials and standard ultrasound machine settings.

Dhir showed a high success rate and patient satisfaction with perineural catheter placement using an agitated solution and color Doppler ultrasound; however, correlation was not made with the agitation coverage pattern (Dhir & Ganapathy, 2008). An in vitro study demonstrated that the injection of a small volume of air (1-2 mL) through a catheter compared to chance improved an expert clinician’s assessment of catheter tip position; however, there was no statistical difference when compared to identifying the catheter position with ultrasound scanning alone or when the test was conducted with a novice practitioner (Kan et al., 2013; Johns et al., 2014). Elsharkawy recommended placing a guide wire through a catheter after it is positioned and then moving the guide wire rapidly inward and outward (pumping maneuver) to create microbubbles for visualization of the catheter track and tip with color Doppler ultrasound (Elsharkawy et al., 2015). However, this study is the first to show an association between the degree of agitation coverage and the likelihood of block success.

This study is not without its limitations. First, we acknowledge the small sample size. We designed this study as a pilot study with a primary goal being feasibility. Secondly, because this study was a feasibility study, we excluded classes 3 and 4 obesity as interpretation of ultrasound is usually more difficult in larger patients. However, we included class 1 obesity as we felt this would strike a balance between generalizability and feasibility. Future studies with larger sample sizes should incorporate individuals with class 2 and higher obesity levels. Many of the blocks that failed to show the agitated injectate around the sciatic nerve still resulted in successful blockade. The mechanism for this is unclear but might be related to proximal or distal migration of the catheter tip during placement. Though anesthesiologists were asked to find the location closest to the catheter prior to injecting the agitated local anesthetic, it is possible that positioning of the ultrasound probe affected the proportion of agitated injectate seen around the nerves. Finally, technologic advancements such as three dimensional ultrasound (Feinglass et al., 2007) or echogenic catheters (Brookes et al., 2015) may improve our capacity to determine catheter tip location; however, the possible economic cost of these technologies may not make them feasible for every clinical practice.

## Conclusion

In conclusion, we demonstrate that injection of an agitated solution through a popliteal sciatic PNC assisted in confirmation of accurate catheter placement and successful blockade when partial or complete coverage of the sciatic nerve or its branches were visualized by color Doppler ultrasound. The presence of partial or complete coverage of the branches of the sciatic nerve was associated improved propensity-adjusted odds of obtaining greater blockade. However, the lack of agitation visualization was not specific for a failed catheter position. We believe an agitated solution can be a useful tool to validate the position of a peripheral nerve catheter placed under ultrasound guidance.

## Data Availability

Please contact author for data requests

## Abbreviations

PNC: Peripheral nerve catheter
D5W: Dextrose 5% in water
ASA: American Society of Anesthesiology
BMI: Body mass index
